# Supplementary material for: Alkaloid Profiling, Anti-Enzymatic and Antiproliferative Activity of the Endemic Chilean Amaryllidaceae Phycella cyrtanthoides
Source: Metabolites. 2022 Feb 18;12(2):188. doi: 10.3390/metabo12020188 (PMC8874788; doi:10.3390/metabo12020188)
Supplement: Supplementary file 1 [file metabolites-12-00188-s001.zip › metabolites-1591976-supplementary.pdf]

Supplementary material for the article:

Alkaloid Profiling, Anti-Enzymatic and Antiproliferative Activity of The Endemic Chilean Amaryllidaceae *Phycella cyrtanthoides*

Carlos Fernández-Galleguillos [1,*], Javier Romero-Parra [2], Adrián Puerta [3], José M Padrón [3] and Mario J. Simirgiotis [1,4,*]

[1] Instituto de Farmacia, Facultad de Ciencias, Universidad Austral de Chile, Campus Isla Teja,
Valdivia 5090000, Chile
[2] Departamento de Química Orgánica y Fisicoquímica, Facultad de Ciencias Químicas y Farmacéuticas,
Universidad de Chile, Olivos 1007, Casilla 233, Santiago 6640022, Chile; javier.romero@ciq.uchile.cl
[3] BioLab, Instituto Universitario de Bio-Orgánica Antonio González (IUBO-AG), Universidad de La Laguna, 38206 La Laguna, Spain; apuertaa@ull.es (A.P.); jmpadron@ull.es (J.M.P.)
[4] Center for Interdisciplinary Studies on the Nervous System (CISNe), Universidad Austral de Chile,
Valdivia 5090000, Chile
* Correspondence: carlos.fernandez@uach.cl (C.F.-G.); mario.simirgiotis@gmail.com or mario.simirgiotis@uach.cl (M.J.S.); Tel.: +56-57-2526910 (C.F-G.); Tel.: +56-63-63233257 (M.J.S.)

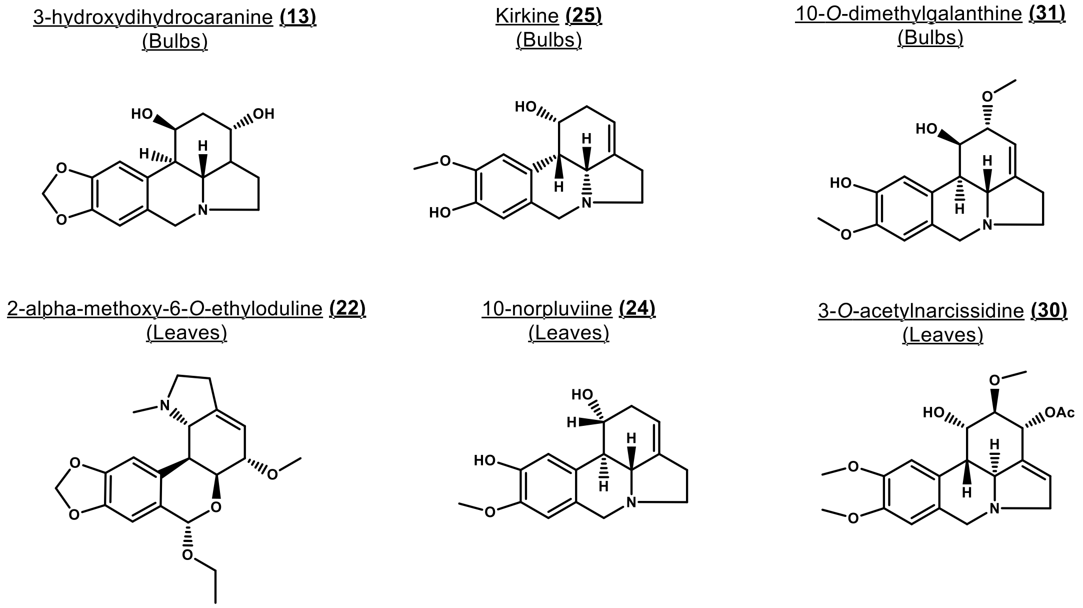

Figure S1. Alkaloids subjected to docking assays in the corresponding catalytic sites of *Torpedo californica* acetylcholinesterase (*Tc*AChE) and human butyrylcholinesterase (*h*BuChE).

Material and Methods

- Docking experiments

Docking experiments were performed using Autodock 4.2 [1]and polar hydrogen atoms of both enzymes were added treating them as rigid bodies. Grid maps were calculated using the autogrid option and were centred on the putative catalytic site of each enzyme considering their known catalytic residues: Ser200 for acetylcholinesterase (TcAChE) [2,3] and Ser198 for butyrylcholinesterase (hBuChE) [4,5] respectively. The volumes chosen for the grid maps were made up of 60 × 60 × 60 points, with a grid-point spacing of 0.375 Å. Docked compound complexes were built using the Lamarckian Genetic Algorithm [6]which involved 100 runs. The lowest docked-energy binding cluster positions were chosen to be analyzed according to the potential intermolecular interactions between inhibitors and the enzymes. The different complexes were visualised in a Visual Molecular Dynamics program (VMD) and Pymol [7].

<u>Results</u>

- Alkaloid profile of *Phycella cyrtanthoides* extracts

The MS/MS spectrum of peak 3, identified as Powelline

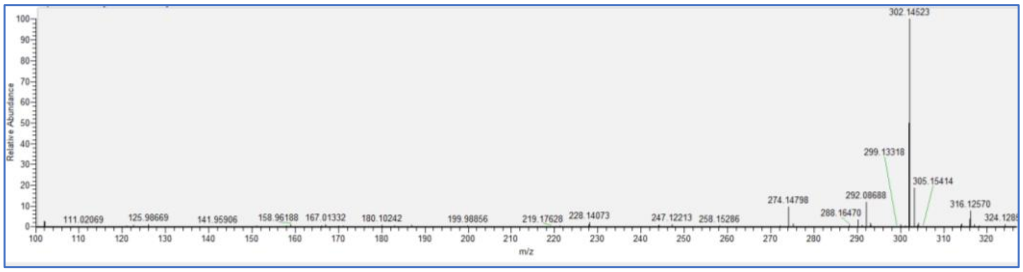

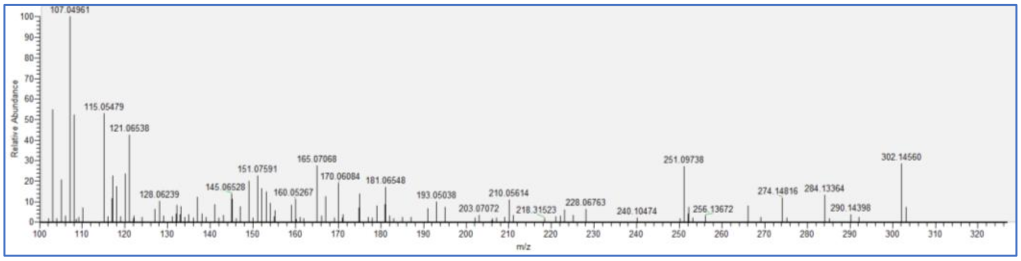

The MS/MS spectrum of peak 4, identified as Lycorine

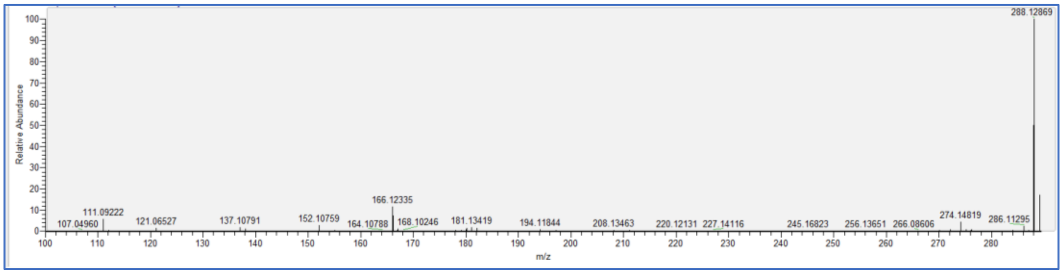

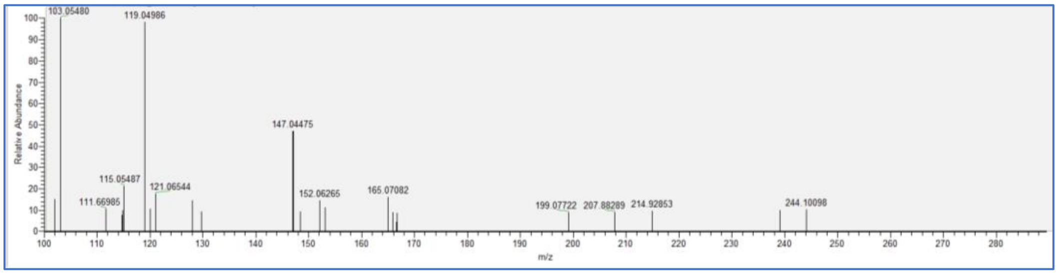

The MS/MS spectrum of peak 6, identified as Hippeastrine

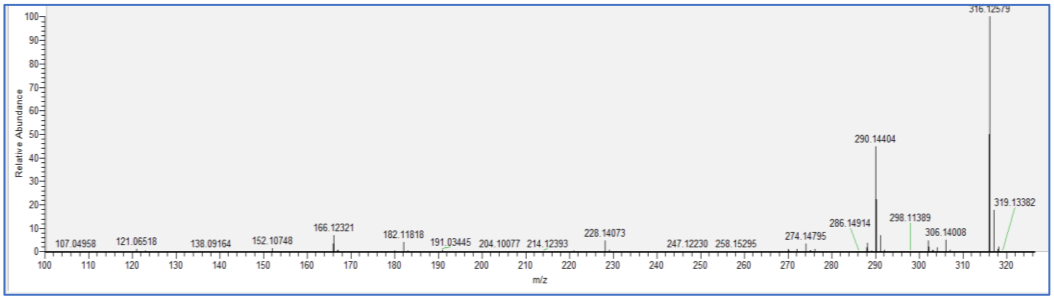

The MS/MS spectrum of peak 20, identified as Haemanthamine

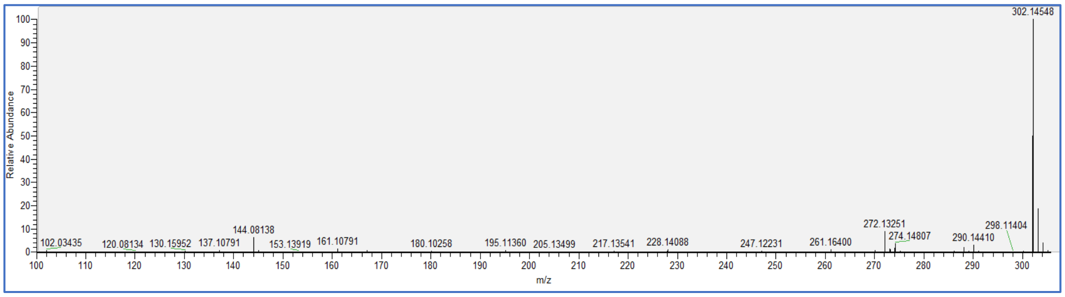

The MS/MS spectrum of peak 23, identified as Vittatine

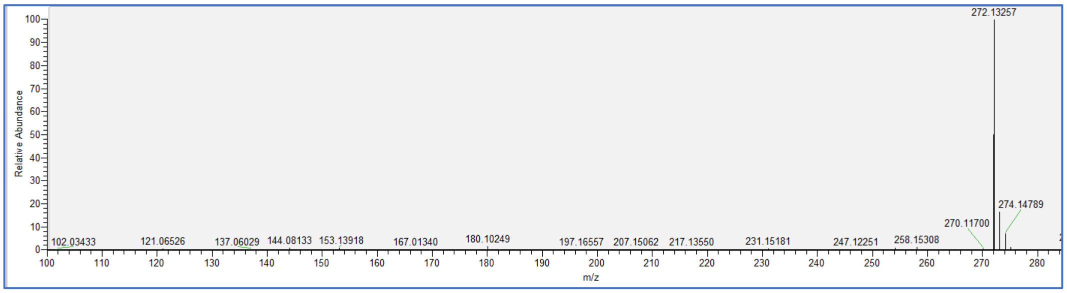

- Antiproliferative effects

Plant organs/cells	A549	HBL-100	HeLa	SW1573	T-47D	WiDr
bulbs	<2,5	<2,5	<2,5	<2,5	<2,5	<2,5
leaves	<2,5	<2,5	<2,5	<2,5	<2,5	<2,5
cisplatin	4.9 ± 0.2	1.9 ± 0.2	1.8 ± 0.5	2.7 ± 0.4	17 ± 3.3	23 ± 4.3

All values were expressed as means ± SEM (n = 3). Cisplatin was used as reference compound

Table S1. Antiproliferative activity (GI_{50}, in μg/mL) of *P. cyrtanthoides*, against human solid tumor cell lines.

References

1. Morris, G.M.; Huey, R.; Lindstrom, W.; Sanner, M.F.; Belew, R.K.; Goodsell, D.S.; Olson, A.J. AutoDock4 and AutoDockTools4: Automated docking with selective receptor flexibility. *J. Comput. Chem. 30*, 2785–2791.

2. Silman, I.; Harel, M.; Axelsen, P.; Raves, M.; Sussman, J.L. Three-dimensional structures of acetylcholinesterase and of its complexes with anticholinesterase agents. In Proceedings of the Biochemical Society Transactions; Portland Press Ltd, 1994; Vol. 22, pp. 745–749.

3. Sussman, J.L.; Harel, M.; Frolow, F.; Oefner, C.; Goldman, A.; Toker, L.; Silman, I. Atomic structure of acetylcholinesterase from *Torpedo californica*: A prototypic acetylcholine-binding protein. *Science (80-.).* **1991**, *253*, 872–879.

4. Nicolet, Y.; Lockridge, O.; Masson, P.; Fontecilla-Camps, J.C.; Nachon, F. Crystal structure of human butyrylcholinesterase and of its complexes with substrate and products. *J. Biol. Chem. 278*, 41141–41147.

5. Tallini, L.; Bastida, J.; Cortes, N.; Osorio, E.; Theoduloz, C.; Schmeda-Hirschmann, G. Cholinesterase Inhibition Activity, Alkaloid Profiling and Molecular Docking of *Chilean Rhodophiala* (Amaryllidaceae). *Molecules* **2018**, *23*, 1532.

6. Thomsen, R.; Christensen, M.H. MolDock: a new technique for high-accuracy molecular docking. *J. Med. Chem. 49*, 3315–3321.

7. DeLano, W.L., The PyMOL molecular graphics system. http://www. pymol. org, 2002.
